# Clinical characteristics and prognosis of sudden sensorineural hearing loss in single-sided deafness patients

**DOI:** 10.3389/fneur.2023.1230340

**Published:** 2023-09-27

**Authors:** Yupeng Liu, Wenjin Wu, Shuna Li, Qing Zhang, Jingchun He, Maoli Duan, Jun Yang

**Affiliations:** ^1^Department of Otorhinolaryngology-Head & Neck Surgery, Xinhua Hospital, Shanghai Jiaotong University School of Medicine, Shanghai, China; ^2^Shanghai Jiaotong University School of Medicine Ear Institute, Shanghai, China; ^3^Shanghai Key Laboratory of Translational Medicine on Ear and Nose Diseases, Shanghai, China; ^4^Ear Nose and Throat Patient Area, Trauma and Reparative Medicine Theme, Karolinska University Hospital, Stockholm, Sweden; ^5^Division of Ear, Nose and Throat Diseases, Department of Clinical Science, Intervention and Technology, Karolinska Institutet, Stockholm, Sweden

**Keywords:** sudden sensorineural hearing loss, single sided deafness, clinical feature, prognosis, glucocorticoid

## Abstract

**Background:**

Sudden sensorineural hearing loss (SSNHL) in patients with single-sided deafness (SSD) is rare. The prognosis of the sole serviceable hearing ear is very important for these patients. However, the clinical characteristics and prognosis of SSNHL in SSD patients are not well-documented.

**Objective:**

This study aimed to investigate the clinical features and treatment outcomes of SSNHL in SSD patients.

**Methods:**

Clinical data of 36 SSD patients and 116 non-SSD patients with unilateral SSNHL from January 2013 to December 2022 were retrospectively investigated. The clinical characteristics of the SSD patients were analyzed. All SSD patients were treated with intratympanic steroids plus intravenous steroids. Pure-tone average (PTA) and word recognition score (WRS) before and after treatment were recorded. The hearing recovery of SSNHL in SSD patients in comparison with non-SSD patients was explored. Auditory outcomes in SSD patients with different etiologies were also compared.

**Results:**

Initial hearing threshold showed no significant differences between the SSD group and the non-SSD group (66.41 ± 24.64 dB HL vs. 69.21 ± 31.48 dB HL, *p* = 0.625). The SSD group had a higher post-treatment hearing threshold (median (interquartile range, IQR) 53.13(36.56) dB HL) than the non-SSD group (median 32.50(47.5) dB HL, *p* < 0.01). Hearing gains (median 8.75(13.00) dB) and the rate of significant recovery (13.89%) were lower in the SSD group than in the non-SSD group (median 23.75(34.69) dB, 45.69%). The etiology of SSD was classified as SSNHL, special types of infection, chronic otitis media, and unknown causes. SSNHL accounted for the maximum proportion (38.9%) of causes of SSD in the SSD group. Hearing gains were lower in the SSNHL-SSD group than in other causes of the SSD group. A binary logistic regression analysis demonstrated that SSD serves as an indicator of unfavorable hearing recovery outcomes (OR = 5.264, *p* < 0.01).

**Conclusion:**

The prognosis of SSNHL in SSD patients is unsatisfactory. SSNHL accounts for the maximum proportion of causes of SSD in this group of patients. For SSD patients caused by SSNHL, less hearing improvement after treatment was expected when SSNHL occurred in the contralateral ear in comparison with SSD patients with other causes.

## Introduction

Sudden sensorineural hearing loss (SSNHL) is characterized by an abrupt onset of sensorineural hearing impairment, involving a decrease of at least 30 dB in hearing across three consecutive frequencies within a span of 72 h. This condition affects an estimated 5 to 20 individuals out of every 100,000 people annually (1). The precise cause of SSNHL remains elusive, but it is widely believed to result from a complex interplay of factors. These factors include viral infections, autoimmune disorders, and vascular insufficiency. The prognosis of SSNHL is influenced by a variety of determinants, which encompass the severity of auditory impairment, the time interval between the onset of symptoms and treatment, and the coexistence of underlying comorbidities (2).

Single-sided deafness (SSD) is defined as a hearing loss of 70 dB or greater in the affected ear, with normal hearing in the other ear (3). In the United States, approximately 7.20% of adults are affected by SSD, with approximately 60,000 new cases emerging each year. Similarly, the United Kingdom reports an annual count of approximately 7,500 new SSD cases (4–6). The underlying etiology of congenital SSD remains elusive, though genetic factors are considered to be primary contributors. In cases of acquired SSD, SSNHL stands as the most frequent factor. Other etiologies encompass head injuries, Meniere’s disease, labyrinthitis, unilateral acoustic neuroma, complications following middle ear surgery, exposure to ototoxic drugs, viral infections, noise-induced hearing loss, and presbycusis. Usami et al. revealed that SSNHL accounted for the majority (54.6%) of cases of post-lingual SSD, followed by various forms of chronic otitis media (7). A subgroup of individuals initially experiencing unilateral profound SSNHL eventually transition to SSD status due to inadequate treatment outcomes. SSD can have a significant impact on the quality of life of affected individuals, including difficulty in localizing sounds, understanding speech in noisy environments, and feeling socially isolated (8, 9).

SSNHL is a debilitating condition that can profoundly affect a patient’s quality of life. In cases where it occurs in individuals with SSD, it presents a distinctive challenge. The prognosis of the remaining functional hearing ear becomes crucial, as any hearing impairment in that ear can significantly compromise their quality of life. Despite the absence of a standardized treatment protocol for SSNHL, glucocorticoids (GCs) have emerged as a foundational pharmacotherapy. GC delivery methods are categorized into systemic and local administration. Systemic administration includes intravenous and oral routes, while local administration commonly involves intratympanic (IT) injections and retroauricular injections. Treatment strategies involving GC encompass both single-agent therapy and combination therapy. However, studies prospectively comparing the effectiveness of different drug delivery methods are scarce. Combination therapy has shown promise in effectively treating severe to profound SSNHL (10, 11). While extensive research has explored the prognosis of SSNHL overall, there is a lack of comprehensive documentation regarding the clinical characteristics and treatment outcomes of SSD patients specifically. Therefore, the objective of this study is to investigate the clinical attributes and treatment responses of SSNHL in individuals with SSD. A comparative analysis will be conducted with non-SSD individuals affected by unilateral SSNHL. Gaining insight into the distinctive features and outcomes of SSNHL within these distinct populations is vital for optimizing management strategies and enhancing treatment outcomes.

## Materials and methods

### Patients

This was a retrospective study that included 36 SSD patients and 116 non-SSD patients with unilateral SSNHL treated at Xinhua Hospital from January 2013 to December 2022. The study was approved by the Institutional Review Board. The diagnostic criteria for SSNHL are defined as a rapid onset of hearing loss, occurring within 72 h, with a sensorineural hearing loss of at least 30 dB in three contiguous frequencies on pure-tone audiometry. These criteria were established by the American Academy of Otolaryngology-Head and Neck Surgery in 2012 (12). The diagnostic criteria for SSD are defined as pure-tone audiometry testing showing a pure-tone average (PTA) of 25 dB HL or greater in the better ear and a PTA of 70 dB HL or greater in the affected ear (3). Exclusion criteria applied in this study encompassed patients with a history of previous otologic surgery, ototoxic drug use, a history of genetic disorders associated with familial deafness, head trauma, retrocochlear disease, and abnormal findings in the central nervous system. Additionally, patients with incomplete medical records or those who did not complete the full course of treatment were excluded from the analysis. Clinical data of all patients were collected, including age, gender, etiology of SSD, hearing thresholds before and after treatment, and treatment methods. The etiology of SSD was determined based on the patients’ medical history, physical examination, laboratory tests, and imaging studies.

### Treatment methods

All patients received a combination treatment of intratympanic steroid (ITS) and intravenous steroid (IVS). The treatment protocol involved a regimen of 10 consecutive days during which patients received intravenous administration of 10 mg dexamethasone, along with IT injections of 2 mg dexamethasone. The successful completion of this 10-day protocol marked the conclusion of the entire treatment course.

### Outcome assessment

The primary outcomes assessed in this study were the changes in pure-tone average (PTA) and word recognition score (WRS) before and after the treatment. Mandarin speech test materials (MSTMs) were utilized for conducting the WRS evaluation. The MSTMs comprised 12 sets of lists, with each list containing 20 sentences. Each sentence consisted of 10 Chinese characters. The testing procedure was conducted using the bilateral implant test (BLIMP) system, maintaining a sound level set at 30 dB above the PTA threshold. A comprehensive test sheet was played, encompassing a total of 20 sentences, each comprising 10 words. WRS was calculated based on this test. The degree of hearing improvement was determined by assessing the alteration in the PTA following the treatment. Given the absence of an “unaffected ear” in the SSD group, a combined approach of the American and Chinese guidelines was employed for outcome assessment (13). Hearing gain ≥30 dB HL was considered indicative of significant recovery. Hearing gain ≥10 dB HL but less than 30 dB HL, or an enhancement in WRS by ≥10% (within the serviceable range, WRS ≥ 50%), was categorized as partial recovery. Hearing improvement of less than 10 dB HL was defined as no recovery. To facilitate the binary logistic regression during the statistical analysis, instances of significant and partial recovery were amalgamated into a “good recovery” category. Conversely, cases of no recovery were categorized as “poor recovery.”

### Statistical analysis

The statistical analysis was conducted using SPSS version 20.0 (IBM Corp., Armonk, NY, United States). Descriptive statistics were utilized to summarize the demographic and clinical characteristics of the study participants. For normally distributed values, the results were presented as mean ± standard deviation. Non-normally distributed values were expressed as median (interquartile range, IQR), while categorical variables were represented as frequency and percentage. To compare continuous variables between the SSD and non-SSD groups, an independent t-test was employed. Categorical variables were compared using the chi-square test. Non-parametric statistics were compared between the two groups using the Mann–Whitney test. Spearman’s correlation analysis was utilized to establish relationships between non-parametric statistics in the two groups. For comparisons among multiple subgroups, non-parametric statistics were analyzed using the Kruskal–Wallis test. A binary logistic regression analysis was performed to calculate the odds ratio (OR) and its corresponding 95% confidence intervals (CIs). This analysis aimed to identify independent prognostic factors associated with SSNHL. The level of statistical significance was defined as a value of *p* of <0.05.

## Results

### Demographics

**Figure 1:**
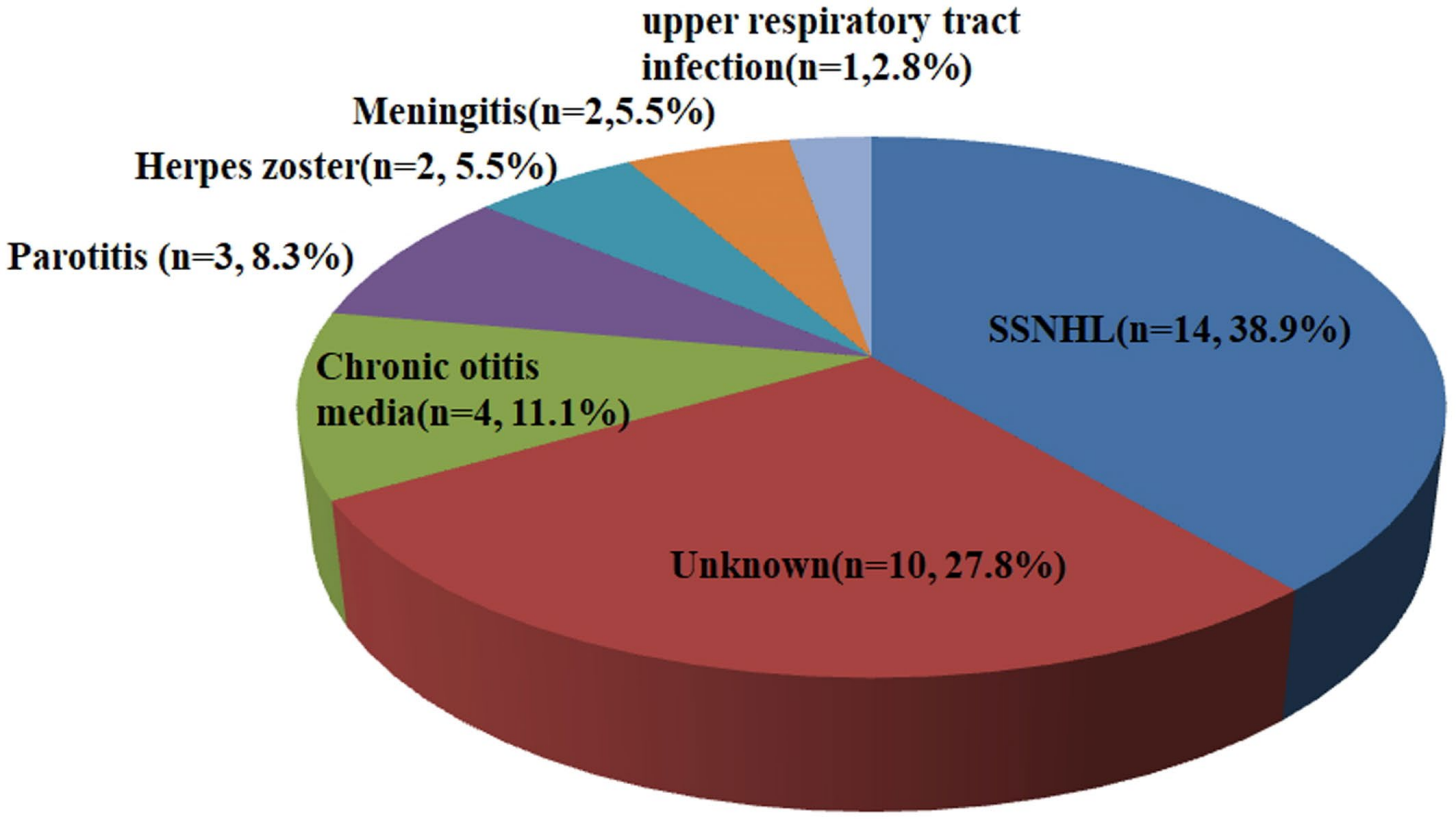
Etiology of hearing loss in the SSD group. Fourteen cases were attributed to SSNHL, eight cases were attributed to special types of infection (three cases of parotitis, two cases of herpes zoster, two cases of meningitis, and one case of upper respiratory tract infection), four cases were attributed to chronic otitis media, and ten cases were attributed to unknown causes. SSD, single-sided deafness; SSNHL, sudden sensorineural hearing loss.

**Figure 2:**
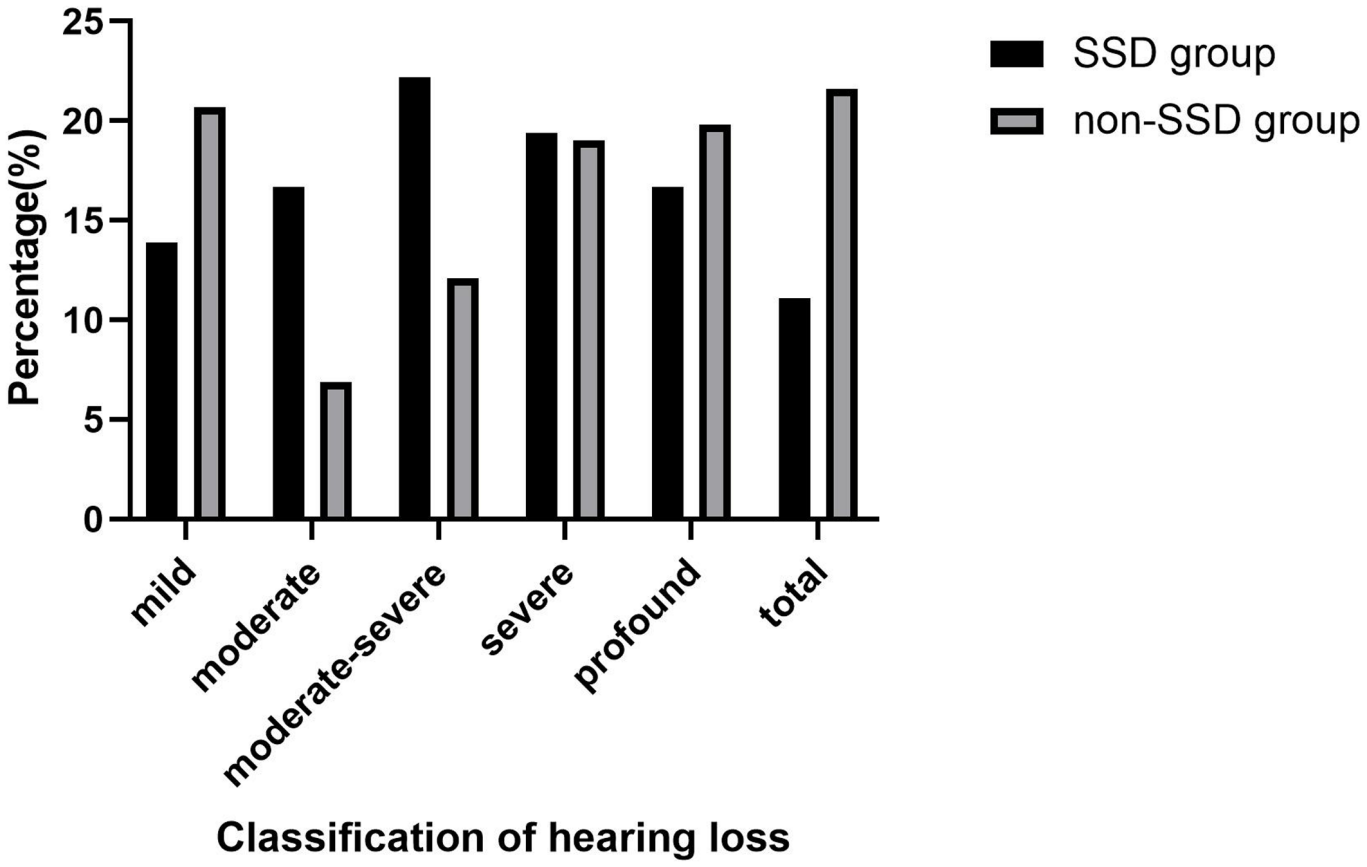
According to the “World Report On Hearing” of the World Health Organization in 2021, hearing loss was classified from “mild” to “total.” In the SSD group, five (13.9%) patients were mild hearing loss, six (16.7%) patients were moderate hearing loss, eight (22.2%) patients were moderate–severe hearing loss, seven (19.4%) patients were severe hearing loss, six (16.7%) patients were profound hearing loss, and four (11.1%) patients were total hearing loss. In the non-SSD group, 24 (20.7%) patients were mild hearing loss, 8 (6.9%) patients were moderate hearing loss, 14 (12.1%) patients were moderate–severe hearing loss, 22 (19.0%) patients were severe hearing loss, 23 (19.8%) patients were profound hearing loss, and 25 (21.6%) patients were total hearing loss. SSD, single-sided deafness.

Table 1 displays the demographic and clinical characteristics of both the SSD and non-SSD groups. The SSD group comprised 20 men and 16 women, with a median age of 59.50 (14.75) years. The affected ear in the SSD group exhibited a median PTA of 97.50 (39.38) dB HL. The median duration of SSD was 8.50 (9.00) years. The non-SSD group consisted of 54 men and 62 women, with a median age of 57.50 (22.75) years. No significant differences were observed in age and gender distribution between the two groups (*p* < 0.05). Additionally, no statistically significant differences were noted in terms of the affected ear, the presence of tinnitus, vertigo, diabetes mellitus, or hypertension. However, there was a statistically significant difference in the interval between symptom onset and treatment initiation, with the SSD group having a median interval of 2.00 (2.00) days compared to 2.00 (5.00) days in the non-SSD group (*p* < 0.05). The initial hearing threshold did not significantly differ between the SSD group and the non-SSD group (66.41 ± 24.64 dB HL vs. 69.21 ± 31.48 dB HL, *p* = 0.625). Figure 1 illustrates the distribution of the etiology of hearing loss in the SSD group. Within the SSD group, the etiology of SSD was categorized as follows: SSNHL (14 cases), special types of infection (8 cases), chronic otitis media (4 cases), and unknown causes (10 cases). Notably, SSNHL accounted for the largest proportion (38.9%) of SSD cases. According to the World Report on Hearing by the World Health Organization in 2021, hearing loss was categorized from “mild” to “total.” In the SSD group, five (13.9%) patients exhibited mild hearing loss, six (16.7%) had moderate hearing loss, eight (22.2%) displayed moderate–severe hearing loss, seven (19.4%) had severe hearing loss, six (16.7%) had profound hearing loss, and four (11.1%) had total hearing loss. In the non-SSD group, 24 (20.7%) patients had mild hearing loss, 8 (6.9%) had moderate hearing loss, 14 (12.1%) had moderate–severe hearing loss, 22 (19.0%) had severe hearing loss, 23 (19.8%) had profound hearing loss, and 25 (21.6%) had total hearing loss. These findings are presented in Figure 2. Notably, there were no significant differences in hearing loss across different frequencies (500 Hz, 1,000 Hz, 2000 Hz, and 4,000 Hz) within both the SSD group (*p* = 0.95) and the non-SSD group (*p* = 0.99).

**Table 1:** Comparison of clinical features of SSNHL patients between the SSD group and the non-SSD group.

	SSD group (*n* = 36)	Non-SSD group (*n* = 116)	*p*-value
Gender, men/women	20/16	56/60	0.445
Age (y)	59.50 (14.75)	57.50 (22.75)	0.201
Side, right/left	19/17	63/53	0.872
Symptom onset to treatment initiation interval, d	2.00 (2.00)	2.00 (5.00)	0.109
Initial hearing threshold (dB HL)	66.41 ± 24.64	69.21 ± 31.48	0.625
Hearing threshold of SSD side (dB HL)	97.50 (39.38)	/	
Duration of SSD (y)	8.50 (9.00)	/	
Tinnitus, *n* (%)	26 (72.2%)	85 (73.3%)	0.901
Vertigo, *n* (%)	9 (25%)	37 (31.9%)	0.431
Hypertension, *n* (%)	10 (27.8%)	19 (16.4%)	0.128
Diabetes, *n* (%)	8 (22.2%)	20 (17.2%)	0.501

SSNHL, sudden sensorineural hearing loss; SSD, single-sided deafness; normal distribution values are mean ± SD; non-normal distribution values are median (interquartile range, IQR).

### Treatment outcomes

**Figure 3:**
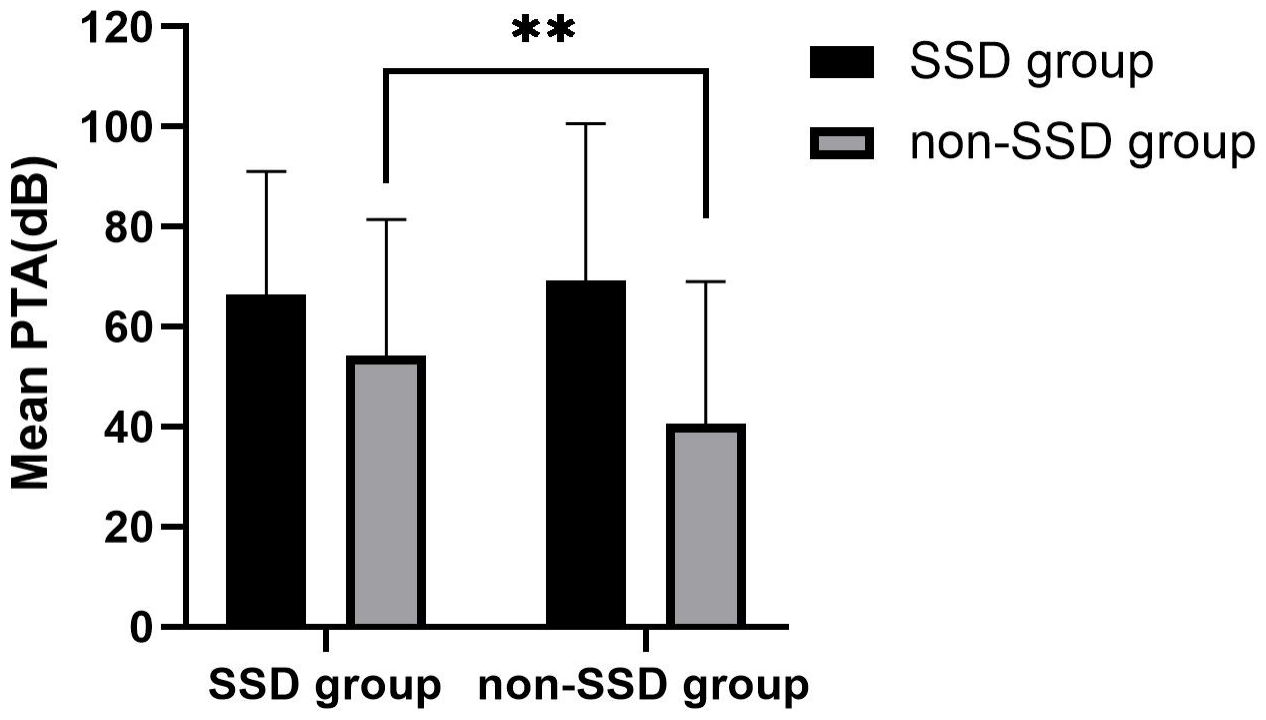
Initial hearing threshold showed no significant differences between the SSD group and the non-SSD group (66.41 ± 24.64 dB HL vs. 69.21 ± 31.48 dB HL, *p* = 0.625). SSD group had a higher post-treatment hearing threshold (median 53.12(36.56) dB HL) than the non-SSD group (32.50(47.50) dB HL, *p* < 0.01). SSD, single-sided deafness.

**Figure 4:**
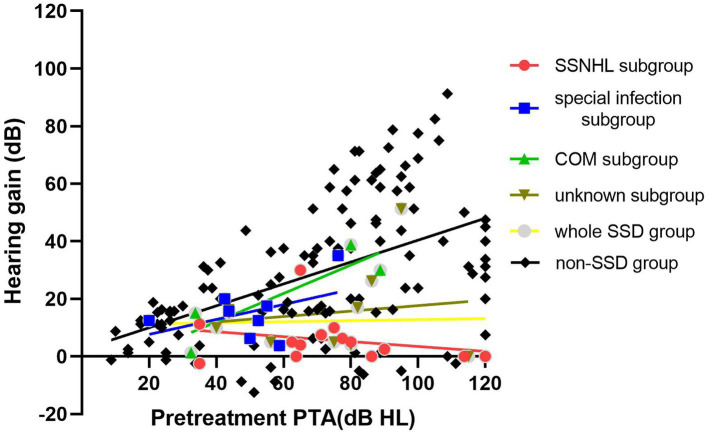
In the whole SSD group and four subgroups, hearing gains were not significantly correlated with pre-treatment PTA (*p* = 0.563, 0.368, 0.866, 0.200, and 0.828, respectively). In the non-SSD group, hearing gains were significantly correlated with pre-treatment PTA (*r* = 0.514, *p* < 0.01). SSD, single-sided deafness; SSNHL, sudden sensorineural hearing loss; COM, chronic otitis media.

Table 2 provides an overview of the treatment outcomes observed in both the SSD and non-SSD groups. Among the patients in the SSD group, 5 individuals (13.89%) showed significant recovery, 13 patients (36.11%) showed partial recovery, and 18 patients (50.0%) showed no recovery. In contrast, within the non-SSD group, 53 patients (45.69%) achieved significant recovery, 38 patients (32.76%) displayed partial recovery, and 25 patients (21.56%) experienced no recovery. The post-treatment hearing threshold was significantly higher in the SSD group (median 53.12 (36.56) dB HL) compared to the non-SSD group (median 32.50 (47.50) dB HL, *p* < 0.01), as depicted in Figure 3. Furthermore, the SSD group exhibited lower hearing gains (median 8.75(13.00) dB) and a decreased rate of significant recovery in contrast to the non-SSD group (median 23.75(34.69) dB). Notably, there were no substantial differences in hearing gains across different frequencies (500 Hz, 1,000 Hz, 2000 Hz, and 4,000 Hz) within either the SSD group (*p* = 0.921) or the non-SSD group (*p* = 0.319). In the SSD group, WRS was 100% in 14 patients prior to treatment. WRS was improved (improvement ≥10%, in the serviceable range) in the remaining 8 of 22 patients. In the non-SSD group, WRS was 100% in 54 patients prior to treatment. WRS was improved (improvement ≥10%, in the serviceable range) in the remaining 43 of 62 patients. The non-SSD group showed a better improvement rate of WRS (*p* = 0.006). Spearman’s correlation analysis was conducted to examine the correlations between pre-treatment PTA and hearing gains in the SSD group, non-SSD group, and the whole population. In the whole SSD group and four subgroups, hearing gains were not significantly correlated with pre-treatment PTA (*p* = 0.563, 0.368, 0.866, 0.200, and 0.828, respectively). Hearing gains showed no significant correlation with PTA on the SSD side either (*p* = 0.432). In the non-SSD group, hearing gains were significantly correlated with pre-treatment PTA (*r* = 0.514, *p* < 0.01). Hearing gains were also significantly correlated with pre-treatment PTA in the whole population (*r* = 0.417, *p* < 0.01), as depicted in Figure 4.

**Table 2:** Treatment outcomes of the SSD and non-SSD groups.

Outcome	SSD group	Non-SSD group	*p*-value
Significant recovery	5(13.9%)	53(45.7%)	<0.01
Partial recovery	13(36.1%)	38(32.8%)	0.710
No recovery	18(50.0%)	25(21.6%)	0.022
Hearing gains (dB)	8.75(13.00)	23.75(34.69)	<0.01
Posttreatment hearing threshold (dB HL)	53.12(36.56)	32.50(47.50)	<0.01

SSD, single-sided deafness.

### Subgroup analysis of the SSD group

**Figure 5:**
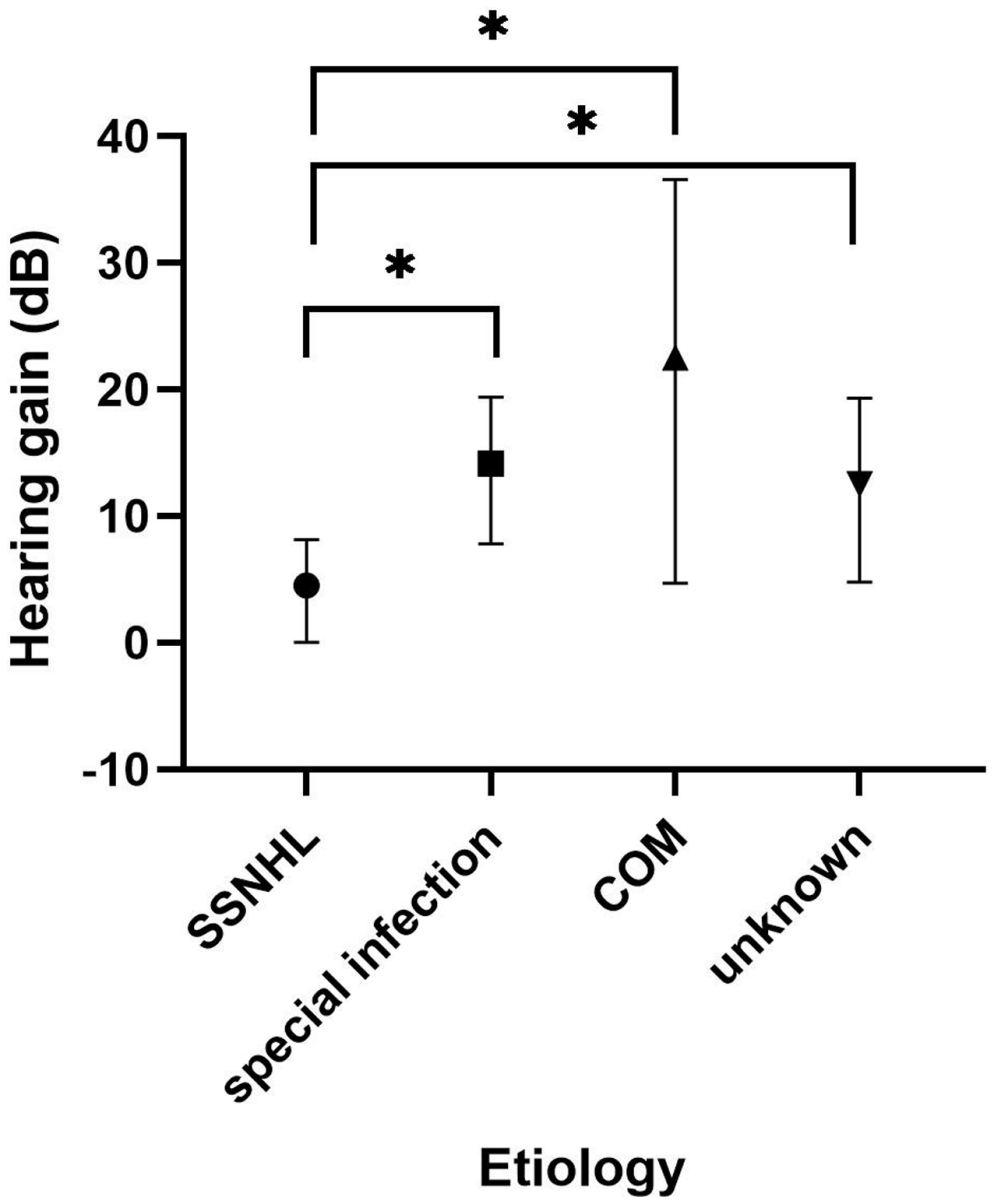
Subgroup analysis of the SSD group based on the cause of SSD. The Kruskal–Wallis test revealed a significant difference between the four groups (*p* = 0.03). The “SSNHL” group had lower hearing gains than the other three groups (*p* = 0.013, 0.034, and 0.048, respectively). SSD, single-sided deafness; SSNHL, sudden sensorineural hearing loss; COM, chronic otitis media.

A subgroup analysis was conducted within the SSD group, categorized based on the cause of SSD. The treatment outcomes of these subgroups are presented in Table 3. In the “SSNHL” subgroup, 1 patient achieved significant recovery, 3 patients showed partial recovery, and 10 patients experienced no recovery. Within the “special infection” subgroup, one patient achieved significant recovery, five patients demonstrated partial recovery, and two patients displayed no recovery. In the “chronic otitis media” subgroup, two patients achieved significant recovery, one patient experienced partial recovery, and one patient had no recovery. In the “unknown cause” subgroup, one patient achieved significant recovery, five patients showed partial recovery, and four patients did not experience recovery. To compare the pre-treatment PTA and hearing gains among the four subgroups, the Kruskal–Wallis test was employed. The results indicated that there was no significant difference in pre-treatment PTA across the four groups (*p* = 0.12). However, a significant difference was observed in terms of hearing gains among the four subgroups (*p* = 0.03). Further analysis using the Steel–Dwass test indicated that the “SSNHL” subgroup had significantly lower hearing gains compared to the other three groups, as illustrated in Figure 5.

**Table 3:** Treatment outcomes of the subgroups of different etiologies.

	SSNHL	Special infection	Chronic otitis media	Unknown
Significant recovery	1 (7.1%)	1 (12.5%)	2 (50.0%)	1 (10.0%)
Partial recovery	3 (21.4%)	5 (61.2%)	1 (25.0%)	5 (50.0%)
No recovery	10 (71.4%)	2 (25.0%)	1 (25.0%)	4 (40.0%)
Hearing gains (dB)	4.50 (8.13)	14.13 (11.57)	22.50 (31.87)	12.50 (14.56)
Posttreatment hearing threshold (dB HL)	64.38 (34.06)	38.75 (19.25)	36.25 (32.50)	55.62 (41.82)

SSNHL, sudden sensorineural hearing loss.

### Prognostic factors of SSNHL

Based on their treatment outcomes, the patients were divided into two groups: good recovery and poor recovery. As a result, 108 patients exhibited good recovery, while 44 patients exhibited poor recovery. Variable comparisons were included in a binary logistic regression analysis. According to the analysis results, symptom onset to treatment initiation interval (OR = 1.125, *p* = 0.016), SSD (OR = 5.264, *p* < 0.01), and diabetes (OR = 2.113, *p* = 0.012) were significantly associated with poor hearing recovery, as outlined in Table 4.

**Table 4:** Clinical factors related to hearing recovery by binary logistic regression.

Variables	OR	95%CI	*p*-value
Age	0.979	0.953–1.006	0.135
Symptom onset to treatment initiation interval, d	1.125	1.022–1.237	0.016
Initial hearing threshold (dB HL)	0.996	0.983–1.010	0.609
SSD	5.264	2.178–12.723	<0.001
Tinnitus	1.342	0.846–2.128	0.219
Vertigo	1.275	0.778–2.076	0.349
Diabetes	2.113	1.182–3.785	0.012
Hypertension	1.464	0.872–2.436	0.150

CI, confidence interval; SSD, single-sided deafness; OR, odds ratio.

## Discussion

Sudden sensorineural hearing loss is a challenging condition that can significantly impact a patient’s quality of life. Its impact becomes particularly intricate when it strikes individuals with SSD, as the prognosis of the remaining functional ear takes on paramount importance. In this retrospective study, we investigated the clinical features and treatment outcomes of SSNHL in SSD patients and compared them with those of non-SSD patients with unilateral SSNHL. Furthermore, we performed a subgroup analysis of the SSD group based on the cause of SSD. Our findings provide valuable insights into the unique characteristics of SSNHL in SSD patients and highlight the importance of optimizing management strategies for this population.

In our study, the results revealed that SSNHL accounted for the maximal proportion (38.9%) of causes of SSD in the SSD group, which is consistent with the previous report. Infectious disease constituted the second largest proportion of the SSD identified in our study. Mumps virus, bacterial meningitis, and herpesvirus are common causes that can lead to unilateral hearing loss (14, 15). Mumps is transmitted through infected respiratory secretions and is highly contagious. The mumps virus directly affects the endolymphatic system of the cochlea, thereby affecting the cochlear spiral organ, the cochlear capsule, and the myelin sheath of the cochlea nerve, leading to hearing loss. Morita et al. reviewed 67 patients with hearing loss caused by a mumps virus infection in a Japanese hospital. Among them, 63 individuals grappled with unilateral hearing loss, with a substantial portion aligning with the criteria for SSD (16). In this study, three patients suffered from bacterial meningitis, which led to SSD in their childhood. Meningitis in infants and young children can cause various complications, with hearing loss being a prominent consequence. Approximately 25% of infants with purulent meningitis will experience long-term hearing loss (17). Among these children, the majority suffer from moderate to severe hearing loss, which can have a serious impact on their quality of life and social interaction abilities.

The importance of treatment efficacy in SSNHL for SSD patients lies in the potential to restore or improve their hearing in the affected ear. Despite several treatments being available, the optimal approach for the treatment of SSNHL remains controversial. Combined IT and systemic GC administration is a promising treatment for SSNHL. Gundogan et al. substantiated the superiority of combined therapy through a prospective, randomized controlled trial (10). In this study, the fourth-week improvements in PTA for the combined therapy group and oral therapy group were 44.05 ± 21.53 dB and 25.72 ± 19.77 dB, respectively. Similarly, Battaglia et al. conducted a multicenter trial to compare hearing recovery outcomes between a combined therapy group and an IT therapy group (11). Their findings underscored that combination therapy provided SSNHL patients with the highest likelihood of achieving class A and B hearing. A recent meta-analysis of randomized controlled trials on the efficacy of combined IT and systemic GC therapies showed that they significantly improved hearing outcomes and increased the recovery rate compared to systemic therapy alone (18). Considering the great importance of hearing recovery for SSD patients, maximal delivery of corticosteroids to the inner ear using both systemic and IT options optimizes the potential for hearing recovery. Although there is still some controversy on the optimal treatment for SSNHL, especially about the efficacy of combination therapy, an aggressive treatment protocol of the combination therapy for SSD group of SSNHL patients is acceptable for both patients and clinicians.

In our study, the initial hearing loss across different frequencies (500 Hz, 1,000 Hz, 2000 Hz, and 4,000 Hz) exhibited no statistically significant differences, both within the SSD group and the non-SSD group. This observation might be attributed to the high prevalence of hearing loss categorized as “severe” or above in both groups (47.2% in the SSD group and 59.5% in the non-SSD group). Patients with hearing loss classified as “severe” to “total” typically display a flat audiogram pattern. Consequently, there is a lack of distinct variations across different frequencies, which could explain the absence of significant differences within these frequencies in our study. Our study also revealed that there was no significant frequency specificity in hearing gains, both within the SSD group and the non-SSD group. This observation contrasts with previous reports that suggest a more favorable hearing recovery for low frequencies compared to high frequencies in SSNHL patients. Suzuki et al. reported that hearing recovery at 500 Hz and 1,000 Hz was notably higher than at other frequencies, and recovery at 8000 Hz was comparatively lower (19). However, Zheng et al. drew a different conclusion in their research. They reported that hearing recovery was significantly greater in the all-frequency SSNHL and total deafness SSNHL subgroups and to a less extent in the low-frequency SSNHL subgroup (20). These disparities in findings could potentially stem from variations in patient characteristics and differences in treatment methodologies. The intricate interplay of these factors likely contributes to the divergence in conclusions observed across different studies.

In this study, the initial hearing thresholds did not exhibit significant differences between the SSD group and the non-SSD group. However, we observed that hearing gains and the rate of significant recovery were notably lower in the SSD group compared to the non-SSD group. To further explore the intricate relationship between SSD and the prognosis of SSNHL, we conducted a binary logistic regression analysis. The outcomes of this analysis revealed that SSD functions as a predictor of unfavorable hearing recovery outcomes (OR = 5.264, *p* < 0.01). This finding underscores the substantial impact of SSD on the potential for hearing improvement in SSNHL cases. The potential mechanism underlying this phenomenon could be associated with deafferentation and subsequent compensatory neural plastic changes occurring within the inferior colliculus. Lee et al. discovered a reduction in the expression of target genes linked to cAMP signaling pathways, metal ion binding, and calcium ion transport within the auditory pathway of SSD rats (21). Moreover, Kim et al. suggested that subcortical auditory neural activities, as observed through Manganum (Mn)-enhanced magnetic resonance imaging, were diminished in regions such as the superior olivary complex, lateral lemniscus, and inferior colliculus on the contralateral side of SSD mice (22). Morphological changes in the cytoskeleton of neurons within the contralateral inferior colliculus were also observed in SSD mice (23). These functional and morphological investigations collectively indicate that both the ipsilateral and contralateral inferior colliculi encounter disruptions within the auditory pathway of SSD patients. Consequently, when patients with healthy ears experience SSNHL, the central auditory system faces pronounced challenges in auditory conduction due to these intricate alterations.

Spearman’s correlation analysis was conducted to examine the relationship between pre-treatment PTA and hearing gains within the SSD group, the non-SSD group, and the whole population. In the SSD group, hearing gains displayed no significant correlation with pre-treatment PTA. In contrast, the non-SSD group showed a significant positive correlation between hearing gains and pre-treatment PTA. This finding seemed to contradict established beliefs that recovery rates decline in proportion to the severity of the initial hearing loss. Prior studies have indicated that the severity of initial hearing loss and audiometric configuration tend to impact prognosis (24–26). However, our results suggested a different perspective. We theorize that this phenomenon may result from the substantial hearing gain experienced by non-SSD patients with profound to total deafness after treatment. In this study, 41.4% of patients in the non-SSD group had profound hearing loss or total deafness. In other words, these individuals had more room for improvement in their hearing levels compared to those with mild to moderate hearing loss. Among the non-SSD group, 66.7% of patients with profound hearing loss or total deafness achieved significant recovery after treatment, while only 20% of patients in the SSD group exhibited significant recovery. This discrepancy suggests that the favorable therapeutic effect observed in severe hearing loss patients within the non-SSD group contributes to the positive correlation between hearing gains and pre-treatment hearing loss. In this study, the lack of a significant correlation between hearing gains and pre-treatment PTA in the SSD group might be linked to the relatively high proportion of “no recovery” patients (50.0%) regardless of their initial hearing loss level. It is noteworthy that the lack of correlation between the PTA on the SSD side and treatment outcomes on the SSNHL side is unexpected. The profound hearing loss already present in the SSD side (97.94 ± 18.49 dB HL) implies a significant level of hearing deprivation that has persisted for years. Given this long-standing condition, the difference between PTA values of 90 dB HL and 100 dB HL becomes essentially negligible, as there is no serviceable hearing. Even if SSD has some influence on the contralateral hearing recovery, this impact appears to be nearly consistent across these patients. Consequently, treatment outcomes show no significant correlations with the PTA on the SSD side.

In our subgroup analysis focusing on the SSD group, the “SSNHL” subgroup stood out by displaying significantly lower hearing gains compared to the other three subgroups. Recurrent cases of SSNHL have been reported to range from 1.4 to 17% in various studies (27). In our study, the patients belonging to the “SSNHL” subgroup could be interpreted as experiencing a second episode of SSNHL in the contralateral ear. The phenomenon of contralateral recurrence in SSNHL patients is relatively uncommon, and the characteristics of this subgroup of patients have not been extensively documented in previous research. The study by Kuo et al. delved into the comparison of two types of recurrence in SSNHL: ipsilateral recurrence and contralateral recurrence. In their investigation of 16 patients, 7 exhibited ipsilateral recurrence, while 9 experienced contralateral recurrence. Their findings revealed no statistically significant differences in the side of recurrence concerning age, inter-episode interval, gender, presence of vertigo, or abnormal caloric results (28). The prognosis for recurrent SSNHL can be quite heterogeneous among individuals. A study by Wu et al. illuminated an interesting relationship between hearing recovery following the first and recurrent episodes of SSNHL. They observed a strong positive association, indicating that a favorable hearing outcome after the initial episode was predictive of a superior outcome after the subsequent episode. Moreover, they identified a distinctive pattern in the distribution of hearing recovery between the first and second episodes. All patients who achieved complete recovery after the second episode also experienced complete recovery after the first episode (29). Obviously, the “SSNHL” group of SSD patients had an unsatisfactory treatment outcome after the first episode. The suboptimal treatment outcomes observed in the “SSNHL” subgroup of SSD patients, both in their first episode and contralateral second attack, may be attributed to the phenomenon of GC resistance. GC resistance in cases of sudden hearing loss refers to the lack of response to standard GC therapy, despite the absence of apparent underlying medical conditions that would hinder a positive response. Overcoming this resistance presents a significant clinical challenge in ensuring effective treatment for patients. Recent research has begun to shed light on potential factors underlying GC resistance in sudden hearing loss. One proposed mechanism involves genetic mutations that impact the expression or activity of GC receptors within the ear. While the exact genetic mechanisms contributing to GC resistance are not fully understood, several genes, including the NR3C1 gene responsible for encoding the GC receptor, have been implicated. Mutations in the NR3C1 gene can lead to altered GC receptor activity or expression, resulting in reduced responsiveness to GC therapy (30). Additionally, GC resistance could be related to decreased expression of histone deacetylase-2, increased levels of macrophage migration inhibitory factor, and P-glycoprotein, along with other factors such as chronic inflammation, oxidative stress, and immune system alterations (31, 32). It is important to recognize that these proposed mechanisms are not mutually exclusive, and it is likely that a combination of factors contributes to GC resistance in sudden hearing loss. Ongoing research aims to further uncover the underlying causes of this resistance and develop more effective treatment strategies for patients who do not respond well to GC therapy. Despite the challenges posed by GC resistance, there are alternative treatment options available for patients in this category. These may involve the use of different medications, such as vasodilators, antioxidants, or anti-inflammatory drugs, as part of a comprehensive approach to managing glucocorticoid-resistant sudden hearing loss. In addition, 21.6% of patients in the non-SSD group experienced “no recovery” following GC therapy. Some of these patients may meet the criteria for SSD based on their hearing levels after treatment. It is crucial for clinicians to fully inform these special patients that if SSNHL occurs again in the contralateral ear, the prognosis for the contralateral ear is generally unfavorable. Consequently, when managing these patients once more, clinicians should not confine themselves to using GC therapy exclusively. They should consider a broader spectrum of treatment options, including vasodilators, antioxidants, and anti-inflammatory drugs. This multifaceted approach ensures comprehensive care tailored to the patient’s unique condition, increasing the likelihood of improved outcomes.

It is important to note that our study has several limitations. First, its retrospective nature and relatively small sample size may restrict the broader applicability of our results. The limited number of participants could potentially introduce bias and affect the robustness of our conclusions. Second, the duration of follow-up in our study was relatively short, which could impede a comprehensive assessment of the long-term outcomes. Extending the follow-up period would offer a more accurate understanding of the prognosis and treatment efficacy over time. Additionally, the treatment protocol employed in our study was based on practices specific to our institution, introducing the possibility of treatment variability across different settings. To address these limitations and enhance the credibility of our conclusions, future research should strive for larger sample sizes, longer follow-up periods, and multi-center collaboration to provide a more comprehensive perspective on the clinical characteristics, treatment outcomes, and management options for SSNHL in SSD patients.

## Data availability statement

The raw data supporting the conclusions of this article will be made available by the authors, without undue reservation.

## Ethics statement

The studies involving humans were approved by Institutional Review Board of Xinhua hospital. The studies were conducted in accordance with the local legislation and institutional requirements. Written informed consent for participation was not required from the participants or the participants’ legal guardians/next of kin in accordance with the national legislation and institutional requirements.

## Author contributions

JH, MD, and JY contributed to the study design. SL and QZ contributed to the statistical analysis and interpretation of the results. YL and WW drafted the manuscript. YL, WW, SL, QZ, JH, MD, and JY revised the manuscript with constructive discussions. All authors contributed to the article and approved the submitted version.

## Funding

This study was supported by the National Natural Science Foundation of China (Nos. 82201280, 82271164, and 82071069).

## Conflict of interest

The authors declare that the research was conducted in the absence of any commercial or financial relationships that could be construed as a potential conflict of interest.

## Publisher’s note

All claims expressed in this article are solely those of the authors and do not necessarily represent those of their affiliated organizations, or those of the publisher, the editors and the reviewers. Any product that may be evaluated in this article, or claim that may be made by its manufacturer, is not guaranteed or endorsed by the publisher.
